# Thyroxine Supplementation in Pregnant Women After Thyroidectomy for Thyroid Cancer and Neonatal Birth Weight

**DOI:** 10.3389/fendo.2021.728199

**Published:** 2021-10-29

**Authors:** Zheng Ding, Fei Guo, Yulai Zhou, Xiaoyi Huang, Zhiwei Liu, Jianxia Fan

**Affiliations:** International Peace Maternity and Child Health Hospital, Shanghai Jiao Tong University, School of Medicine, Shanghai, China

**Keywords:** thyroidectomy, early pregnancy, thyroid cancer (TC), birth weight (BW), thyroxine supplementation

## Abstract

Patients are often supplemented with a sufficient dose of thyroxine after thyroidectomy for thyroid cancer. However, the influence of thyroxine supplementation on fetal growth in pregnant women after thyroidectomy for thyroid cancer remains unclear. The aim of this study was to investigate the effect of thyroxine supplementation on neonatal birth weight. This cohort study included 49,896 pregnant women (278 patients with a history of thyroidectomy for thyroid cancer and 39,363 control cases after exclusion). Thyroid parameters were examined in pregnant women and their newborns. The associations between maternal thyroid function and neonatal birth weight and small for gestational age were studied using regression analyses. In the levothyroxine supplementation group, free thyroxine (FT4) levels were significantly higher in both early pregnancy (*P* < 0.001) and late pregnancy (*P* < 0.001) groups than in the control group. Furthermore, levels of neonatal thyroid stimulating hormone (*P* = 0.032) and birth weight (*P* = 0.043) were significantly lower than those in the control group. We also observed a significant inverse association between maternal FT4 levels in early pregnancy and neonatal birth weight (*P*=0.028), especially in male newborns (*P*=0.036). In summary, after thyroidectomy for thyroid cancer, a sufficient dose of thyroxine supplementation in early pregnancy is significantly associated with reduced birth weight and may need to be monitored.

## Introduction

Thyroid cancer is a common endocrine malignancy in young women. The prevalence of thyroid cancer in pregnancy (14.4/100,000) was reported (1), and 3.3/100,000 was diagnosed before delivery (2). Women of childbearing age are particularly concerned because of their potential influence on the fetus. Women usually undergo thyroidectomy for thyroid cancer. After surgery, thyroid hormone replacement or thyroid stimulating hormone (TSH) suppression therapy with levothyroxine (LT4) to prevent hypothyroidism or tumor recurrence is needed. However, it is often difficult to adjust the dose of the drug to maintain an ideal thyroxine level in clinical practice, especially in pregnant women. To date, few studies have examined the effect of maternal thyroxine supplementation on fetal growth in women with a history of thyroidectomy for thyroid cancer.

Thyroid hormone is a well-known regulator of growth and development in the human body and plays a crucial physiological role in early placental development and intrauterine fetal growth (3, 4). Fetal growth during early pregnancy is a relevant determinant of pregnancy outcomes and later child health. Maternal abnormal thyroid function during pregnancy is associated with a wide range of adverse fetal growth and development, including spontaneous abortion, preterm delivery, neurodevelopmental delay, small head circumference, and low birth weight (5). Even in mothers with normal range FT4 and TSH levels, higher maternal FT4 levels in early pregnancy are associated with lower birth weight and an increased risk of small for gestational age (SGA) at birth (6), demonstrating that even mild variations in thyroid function within the normal range during early pregnancy can have important fetal consequences.

Fetal thyroid hormone production starts around 14th week of pregnancy, and the fetal thyroid gland is functionally mature between the 18th and 20th weeks. Therefore, fetal thyroxine during the first half of pregnancy depends on placental transfer of maternal thyroid hormones (7, 8). In this period, optimal intrauterine conditions are essential for proper fetal development and growth, and a suboptimal intrauterine environment may affect fetal growth (9). A study has shown that overt maternal and fetal thyroid hyperfunction are associated with a reduced birth weight, and fetal thyroid hormone levels at birth are associated with fetal growth (10). Impaired fetal growth is a major predictor of neonatal mortality and morbidity and may increase the risk of long-term health complications, such as diabetes and cardiovascular disease in adulthood (11, 12). Birth weight is the most important indicator of fetal growth and development in the uterus and reflects fetal adaptations to the intrauterine environment (9). However, it is unclear how LT4 supplementation influences fetal growth after maternal thyroidectomy for thyroid cancer, and the study of sufficient thyroxine supplementation in regulating fetal thyroid function and fetal growth is not well established. Therefore, this study aimed to explore the influence of LT4 supplementation on neonatal thyroid function and birth weight in women with a history of thyroidectomy for thyroid cancer.

## Materials and Methods

### Study Population

A total of 49896 pregnant women who delivered in a large specialized hospital for women and children between January 2016 and December 2018 were included in the study. Of these, 278 patients underwent a total thyroidectomy due to papillary thyroid cancer and they required a sufficient dose of LT4 for replacement treatment or the TSH suppression (sufficient dose of LT4 in this study refers to supplementary LT4 that can suppress TSH levels near the lower limit of pregnancy-specific reference range). Data were collected from electronic medical records. Information on thyroid function parameters was available for the included pregnant women. Data were also available on potential confounders, including gestational age at birth, maternal age, parity, education level, pre-pregnancy body mass index (BMI), gestational hypertension, gestational diabetes, medical history, and fetal sex. Alcohol consumption and smoking status were not included in the analysis because their use was rare among pregnant women in our study population. Exclusion criteria for the study population mainly included women who underwent *in vitro* fertilization (IVF) or had a twin pregnancy or had other pre-existing thyroid diseases (including hyperthyroidism, hypothyroidism, thyroiditis, surgery for benign thyroid nodule, and medication use for thyroid disease). The study protocol was approved by the Medical Ethics Committee of the International Peace Maternity and Child Health Hospital. Written informed consent was obtained from all participants.

### Thyroid Hormone Measurements

Maternal serum samples were obtained in the first trimester (11–13 weeks) and third trimester (31-33 weeks) of pregnancy. Fasting blood samples were drawn from the median cubital vein and the serum was separated by centrifugation within 6 h. The levels of TSH, FT4, total triiodothyronine (TT3), and thyroid peroxidase antibody (TPOAb) were measured using the Architect i2000 immunoassay (Abbott, Chicago, IL, USA) according to the manufacturer’s protocol. The TPOAb concentration was considered positive when levels were above 5.61 IU/mL. The reference ranges for TSH, FT4 and TT3 were defined by the 2.5th and 97.5th percentiles of the entire population of pregnant women who gave birth in our hospital between January 2016 and December 2018 after exclusion of twin pregnancies, IVF, women who had other pre-existing thyroid diseases and women with positive TPOAb. Neonatal plantar blood was collected within one week after delivery, and TSH levels were measured according to the manufacturer’s protocol.

### Outcome Measurements

The primary outcome was neonatal birth weight (in grams) of live born, full-term neonates (gestational duration ≥ 37 weeks). Birth weight was measured by midwives and obstetricians attending birth and was studied as a continuous variable. SGA was defined as a gestational age-adjusted birth weight below the 10th percentile in the study cohort. Large for gestational age (LGA) was defined as a gestational age-adjusted birth weight above the 90th percentile in the study cohort. To combine and compare birth weights according to gestational age, we standardized the raw birth weight using *Z* scores based on the gestational age-specific means and standard deviations of the corresponding measurements. An early ultrasound scan of the crown-rump length was used to correct gestational dating when the difference using the last menstrual period was equal to or greater than 7 days. The TSH indices of neonatal plantar blood were measured within one week of birth.

### Study Covariates

Information on covariates was obtained from questionnaires and clinical registries during pregnancy, including gestational age at birth, maternal age, parity, educational level, pregnancy-induced hypertension, gestational diabetes mellitus, and medication use. At the first outpatient visit, maternal height and weight were measured to calculate the BMI (kg/m^2^). Information on fertility treatment and fetal sex was obtained from midwives and obstetricians.

### Statistical Analyses

Continuous and categorical variables were expressed as mean ± standard deviation (SD) and percentages, respectively. Birth weight was adjusted for gestational age at delivery. The differences in maternal thyroid function in the first and third trimesters of pregnancy, as well as neonatal TSH of plantar blood and birth weight were compared using Student’s t-test or Mann-Whitney test. The rates of SGA and LGA were compared between the two groups using the χ^2^ test. Linear regression and multinomial logistic regression models were used to assess the association between thyroid function indices and birth weight and the risk of SGA and LGA, respectively. Model assumptions were evaluated with residual plots, and potential nonlinearity was assessed using three restricted cubic splines. Analyses were adjusted for potential confounders, including gestational age at birth, maternal age, parity, educational level, BMI, gestational hypertension, gestational diabetes, and fetal sex. To evaluate the stability and test the robustness of our findings under the influence of potential confounders, we performed a sensitivity analysis using propensity score matching (PSM, 1:2 ratio) method. After excluding missing values, the research objects were matched between the LT4 supplementation group and the control group according to the scores of potential variables on gestational age at birth, maternal age, BMI, gestational hypertension, gestational diabetes, fetal sex, and maternal thyroid function indices.

All statistical analyses were performed using R statistical software (version 3.6.0; package rms, visreg, mass) or IBM SPSS Statistics for Windows (Version 20.0, IBM Corp., Armonk, NY, USA). Statistical significance was set at *P* < 0.05.

## Results

### Population Characteristics

**Figure 1:**
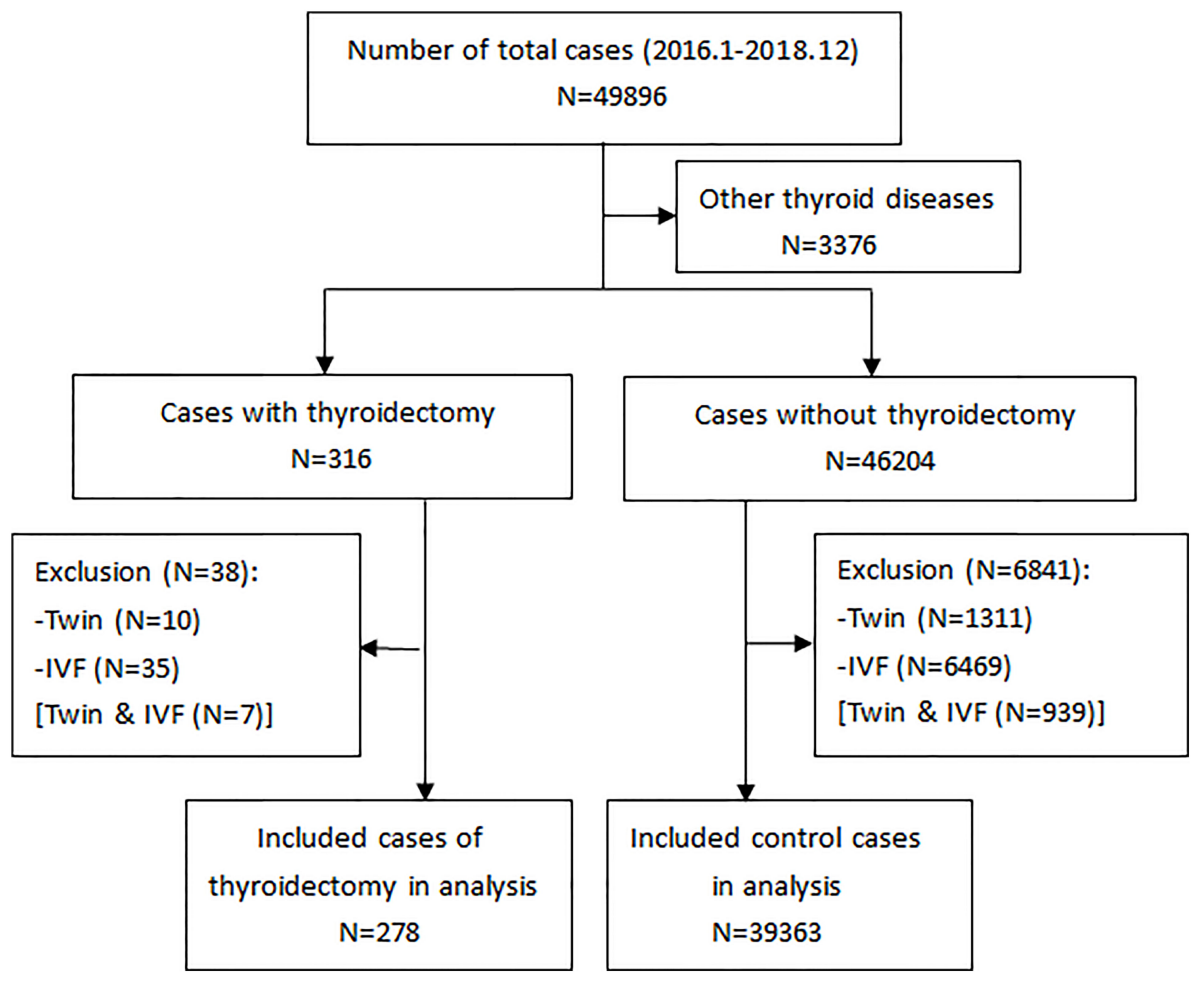
A flowchart of the study selection. Other thyroid diseases (in the second box) include hyperthyroidism, hypothyroidism, thyroiditis, surgery for benign thyroid nodule, and medication use for thyroid disease.

After exclusion of twin pregnancies, IVF, and other pre-existing thyroid diseases, the final study population comprised 278 pregnant women with a history of thyroidectomy for thyroid cancer and 39,363 pregnant women without thyroid surgery (**Figure 1**). Of these, 1877 control women with incomplete thyroid function indices (4.77%) were excluded from subsequent analyses. The baseline descriptive characteristics of the study population are presented in **Table 1**. In the study, the mean (SD) maternal age was 32.3 (4.5) and 30.8 (4.0) years in the LT4 supplementation group and control group, respectively. The rates of gestational diabetes were 13.3% and 11.7%, and the rates of gestational hypertension were 3.2% and 2.7%, respectively. The percentage of TPOAb positivity in the study population was 14.7% and 9.5%, respectively, according to the manufacturer’s cutoff value. Pregnancy-specific reference ranges for TSH, FT4, and TT3 are 0.02–3.6 mU/L, 11.4–18.5 pmol/L, and 1.5–2.9 nmol/L in the first trimester of pregnancy, respectively.

**Table 1:** Baseline characteristics of the study population.

Characteristic	LT4 supplementation (N = 278)	Control (N = 39363)	P-value
Age, Mean (yr)	32.3 ± 4.5	30.8 ± 4.0	<0.001
Gestational age at birth,			
Mean (ws)	38.9 ± 1.5	38. 9 ± 1.4	0.837
Parity, n (%)			<0.001
Primiparous	159 (57.2%)	26713 (67.9%)	
Multiparous	119 (42.8%)	12650 (32.1%)	
Education level, n (%)			0.104
Primary education	38 (13.7%)	7607 (19.3%)	
Bachelor’s	200 (71.9%)	25876 (65.7%)	
Master’s	37 (13.3%)	5195 (13.2%)	
Doctoral	3 (1.1%)	457 (1.2%)	
Pregnancy-induced			
hypertension, n (%)	9 (3.2%)	1072 (2.7%)	0.6
Gestational diabetes			
mellitus, n (%)	37 (13.3%)	4609 (11.7%)	0.408
TPOAb, n (%)			0.001
Negative	214 (77.0%)	34236 (87.0%)	
Positive	41 (14.7%)	3754 (9.5%)	
Body mass index (kg/m^2^)	21.5 ± 2.9	21.1 ± 2.8	0.015
Neonatal sex (%)			0.057
Male	57.2%	51.5%	
Female	42.8%	48.5%	

LT4, levothyrocine; TPOAb, thyroid peroxidase antibody.

All thyroidectomies were performed before pregnancy. The median time from thyroidectomy to pregnancy was 4 years (2-7 years). In this group, the dose of LT4 was determined based on the level of TSH, and the mean LT4 dose was 122.5ug per day (87.5–200ug/d). Some cases increased the dose of LT4 during pregnancy, with the increased dose ranging from 12.5ug to 50ug per day, which is about 12.5%–33.3% increase in the percentage of the initial dose. All pregnant women were administered oral LT4 throughout their pregnancies.

### The Differences in Indices of Maternal Thyroid Function

**Figure 2:**
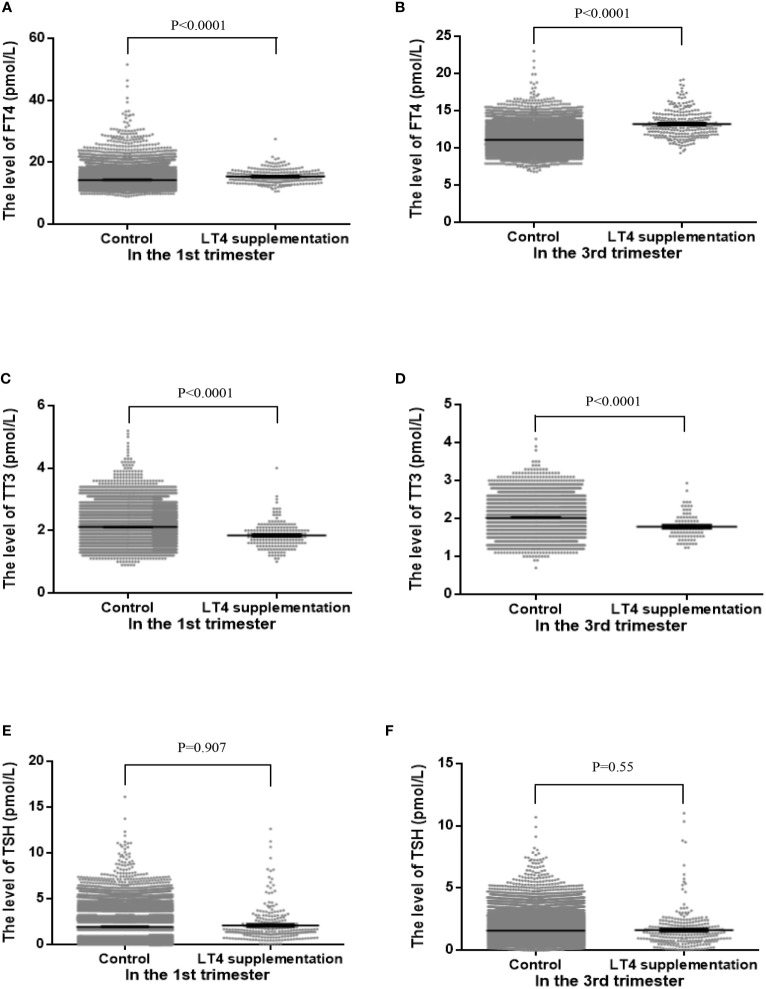
The distributions of maternal free thyroxine (FT4, **A**, **B**), total triiodothyronine (TT3, **C**, **D**) and thyrotropin (TSH, **E**, **F**) levels between control group and LT4 supplementation group in the first trimester (left panels) and third trimester (right panels).

When comparing the differences in the maternal thyroid function indices in the first and third trimesters of pregnancy between the two groups, the FT4 level in the LT4 supplementation group was found to be significantly higher both in the first (mean 15.37 ± 2.08 *vs.* 14.24 ± 1.71, *P* < 0.001) and third trimesters (mean 13.05 ± 1.82 *vs.* 11.1 ± 1.24, *P* < 0.001), and the TT3 level in the LT4 supplementation group was significantly lower both in the first (mean 1.84 ± 0.4 *vs.* 2.12 ± 0.38, *P* < 0.001) and third trimesters (mean 1.76 ± 0.29 *vs.* 2.02 ± 0.36, *P* < 0.001). The mean TSH level in the LT4 supplementation group was not significantly different from the control group. The differences in the maternal thyroid function indices between the two groups are shown in **Table 2** and **Figure 2**.

**Table 2:** Differences in indices of maternal thyroid function between LT4 supplementation group and control group.

Term	LT4 supplementation(N=278)	Control(N=39363)	P-value
FT4, Mean (pmol/L)			
1st trimester	15.37 ± 2.08	14.24 ± 1.71	<0.001
3rd trimester	13.05 ± 1.82	11.1 ± 1.24	<0.001
TT3, Mean(nmol/L)			
1st trimester	1.84 ± 0.4	2.12 ± 0.38	<0.001
3rd trimester	1.76 ± 0.29	2.02 ± 0.36	<0.001
TSH, Mean(mU/L)*			
1st trimester	1.85 ± 1.87	1.88 ± 1.64	0.907
3rd trimester	1.48 ± 1.56	1.57 ± 0.81	0.55

LT4, levothyrocine; FT4, free thyroxine; TT3, total triiodothyronine; TSH, thyroid stimulating hormone. Pregnancy-specific reference ranges for FT4, TT3, and TSH are 11.4-18.5 pmol/L, 1.5-2.9 nmol/L, and 0.02-3.6 mU/L in the first trimester of pregnancy and 8.9-13.7pmol/L,1.4-2.8nmol/L, and 0.4-3.5mU/L in the third trimester of pregnancy, respectively.

*The comparisons of TSH between two groups were performed using Mann-Whitney test due to non-normal distribution of TSH.

### The Differences in Neonatal TSH Levels and Birth Weight

We found a difference in the TSH level of neonatal plantar blood within a week of birth between the two groups, and the mean neonatal TSH level was lower in the LT4 supplementation group than in the control group. The whole neonatal birth weight was significantly lower in the LT4 supplementation group than in the control group (3292 g *vs.* 3372 g, *P*=0.043) (**Table 3**). On sensitivity analysis using the propensity score matching method, 278 pregnant women with a history of thyroidectomy were matched at a ratio of 1:2 with the control women without a history of thyroid disease based on the adjustment for potential confounders possibly associated with abnormal fetal growth. We found that the difference in neonatal TSH level between two groups was significant (mean 1.61 ± 1.26 *vs.* 1.84 ± 1.47, *P* = 0.032). After propensity score matching, there was no significant change in the directionality of the difference in birth weight (3274 g *vs.* 3402 g, *P*=0.01). Subgroup analysis revealed a consistent difference in men before and after propensity score matching. However, there was no significant difference in the risk of SGA between the LT4 supplementation group and the control group (odds ratio [OR], 1.41; 95% confidence interval, CI: 0.59–3.35, *P*= 0.461). The results are presented in **Table 3**.

**Table 3:** Differences of neonatal outcomes before and after propensity score matching.

Outcomes	Before Matching			After Matching		
	LT4 supplementation (N = 278)	Control (N = 39363)	P-value	LT4 supplementation (N = 250)	Control (N = 500)	P-value
Neonatal TSH(mU/L)						
	1.72 ± 1.43	1.80 ± 2.01	0.597	1.61 ± 1.26	1.84 ± 1.47	0.032
Birth weight(g)						
Total	3292 ± 444.4	3372.2 ± 389.5	0.043	3273.6 ± 460.2	3401.6 ± 383.9	0.01
Male	3363.9 ± 448.4	3475 ± 391.2	0.033	3333.1 ± 467	3470.5 ± 402.4	0.045
Female	3179.8 ± 418.1	3316.9 ± 379.9	0.025	3172.8 ± 435.9	3302.1 ± 333.2	0.105
SGA, n(%)	11 (3.96)	1240 (3.15)	0.815	11 (4.4)	10 (2.0)	0.282
SGA (OR[CI])*	1.41 [0.59-3.35]		0.461	1.53 [0.54-3.41]		0.437

LT4, levothyrocine; SGA, small for gestational age.

*OR, odds ratio; CI, confidence interval. Adjusting for gestational age at birth, maternal age, body mass index, parity, educational level, pregnancy-induced hypertension, gestational diabetes mellitus, and fetal sex.

### Association of Maternal Thyroxine With Neonatal Birth Weight

**Figure 3:**
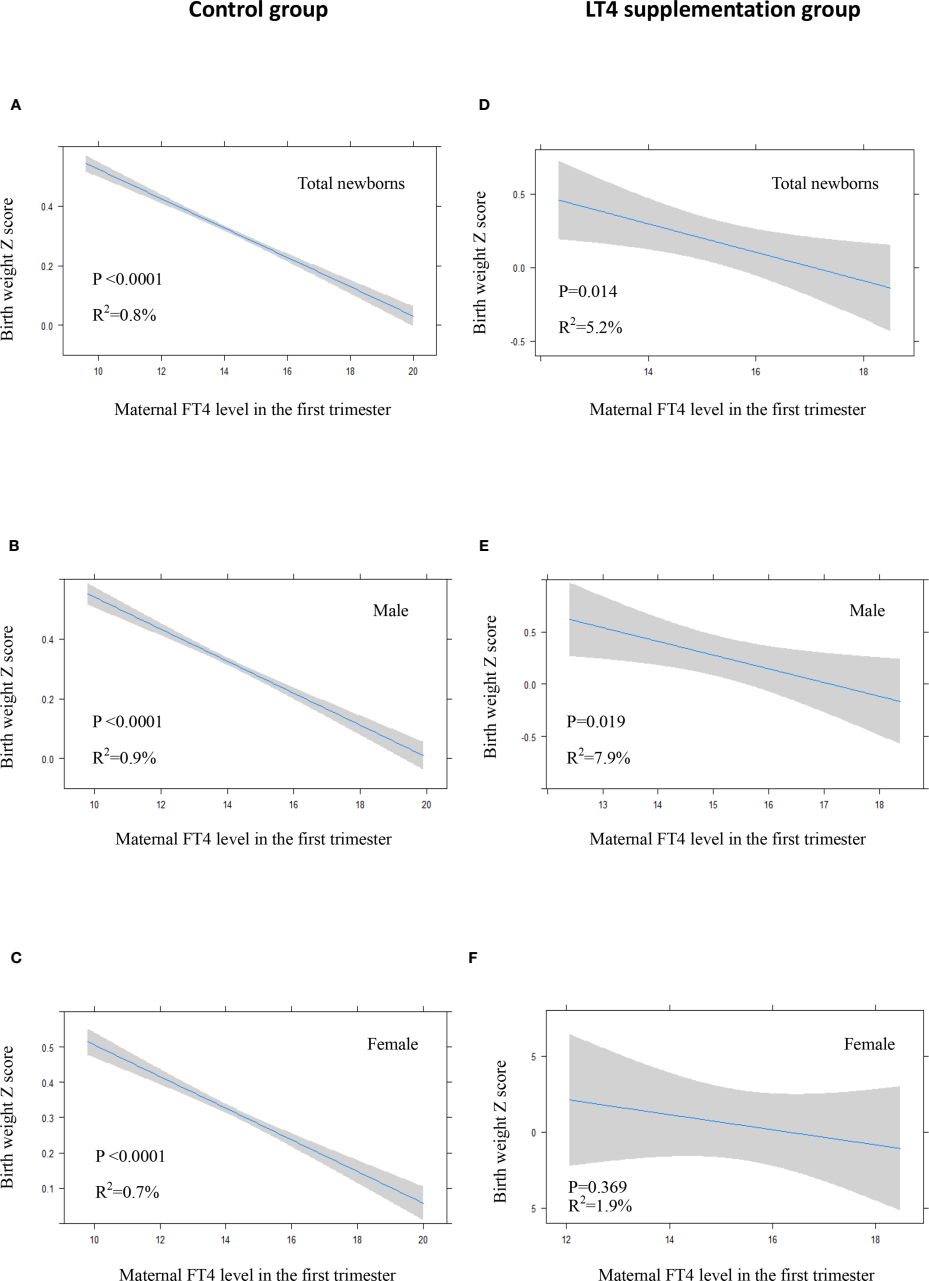
In crude model: Plots show the association of maternal FT4 level in the first trimester with birth weight of total newborns **(A, D)**, male newborns **(B, E)** and female newborns **(C, F)** in control group **(A–C)** and LT4 supplementation group **(D–F)** as predicted mean (blue line) with 95% confidence interval (CI, gray area). The neonatal birth weight was standardized for Z score.

**Figure 4:**
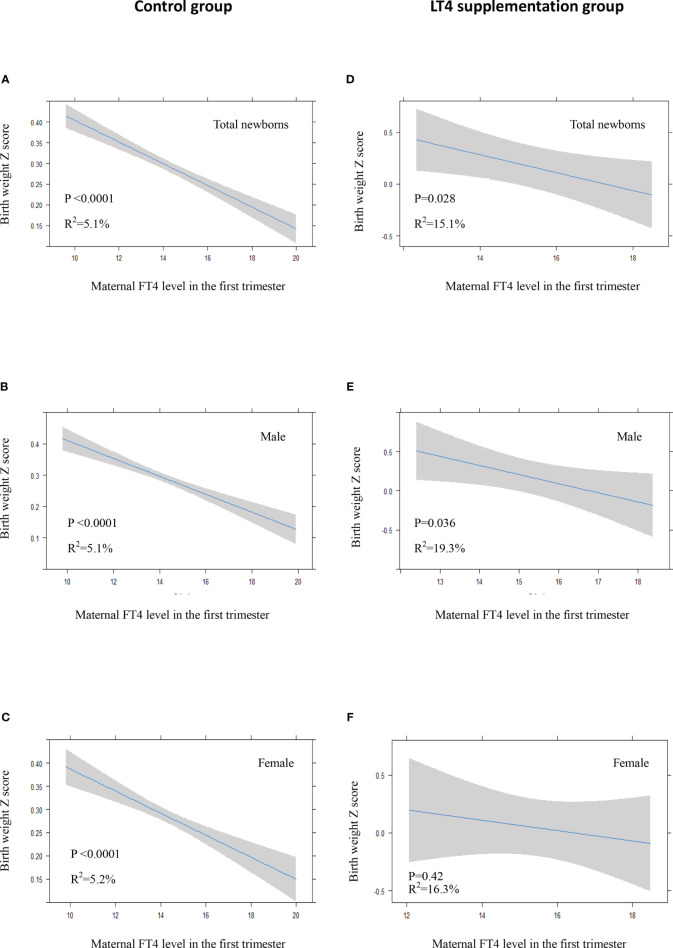
In adjusted model: Plots show the association of maternal FT4 level in the first trimester with birth weight of total newborns **(A, D)**, male newborns **(B, E)** and female newborns **(C, F)** in control group **(A–C)** and LT4 supplementation group **(D–F)** as predicted mean (blue line) with 95% confidence interval (CI, gray area). The neonatal birth weight was standardized for Z score. Analyses were adjusted for gestation age at birth, maternal age, body mass index (BMI), pregnancy-induced hypertension (PIH), gestational diabetes mellitus (GDM), and fetal sex.

There was a significant inverse association between maternal FT4 levels in the first trimester and neonatal birth weight in the control group either in crude model or in adjusted model (**Figures 3A–C**, **4A–C**). We also observed a significant inverse association between maternal FT4 levels in the first trimester and neonatal birth weight in the LT4 supplementation group (β = -0.1; 95% CI,[-0.17~ -0.02]; *P*=0.014) (**Table 4**). A higher maternal FT4 level in the first trimester of pregnancy was associated with a lower neonatal birth weight, with an estimated effect of approximately 0.6 Z score across the full range of FT4 (**Figure 3D**). Furthermore, the association between maternal FT4 in the first trimester and birth weight was more significant in male newborns (β = -0.13; 95% CI, [-0.24~ -0.02]; *P*=0.019) (**Figure 3E**). The associations remained significant after further adjustment for potential confounders, including gestational age at birth, maternal age, BMI, gestational hypertension, gestational diabetes, and fetal sex (**Figures 4D, E**). However, there was no difference in female newborns in the FT4 supplementation group in either the crude model or the adjusted model (**Figures 3F** and **4F**).

**Table 4:** Association of maternal FT4 in the first trimester with birth weight.

Term	LT4 supplementation group		Control group	
	β [95%CI]	P	β [95%CI]	P
Crude model				
Total	-0.1 (-0.17,-0.02)	0.014	-0.05 (-0.06,-0.04)	<0.0001
Male	-0.13 (-0.24,-0.02)	0.019	-0.05 (-0.06,-0.05)	<0.0001
Female	-0.05 (-0.16,0.06)	0.369	-0.05 (-0.05,-0.04)	<0.0001
Adjusted model				
Total	-0.09 (-0.16,-0.01)	0.028	-0.03 (-0.03,-0.02)	<0.0001
Male	-0.12 (-0.23,-0.01)	0.036	-0.03 (-0.04,-0.02)	<0.0001
Female	-0.05 (-0.16,0.07)	0.42	-0.02 (-0.03,-0.02)	<0.0001

FT4, free thyroxine; LT4, levothyrocine; CI, confidence interval.

Crude model: without adjustment for potential confounders.

Adjusted model: analyses were adjusted for gestation age at birth, maternal age, body mass index (BMI), pregnancy-induced hypertension (PIH), gestational diabetes mellitus (GDM), and fetal sex.

### Association of Maternal TSH With Neonatal Birth Weight

**Figure 5:**
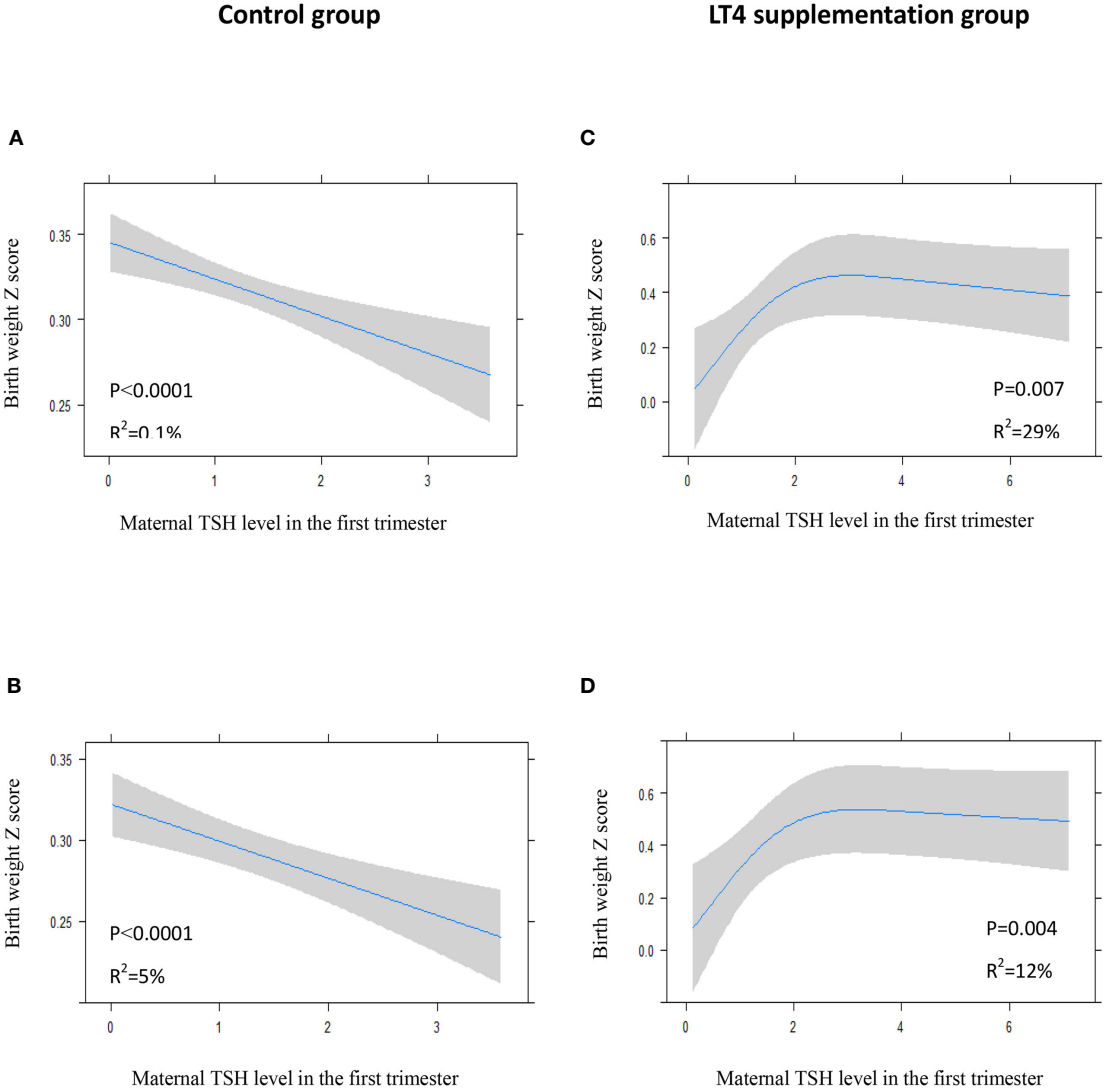
Plots show the association of maternal TSH level in the first trimester with birth weight of total study newborns in control group **(A, B)** and LT4 supplementation group **(C, D)** as predicted mean (blue line) with 95% confidence interval (CI, gray area). The neonatal birth weight was standardized for Z score. Crude model **(A, C)**: without adjustment for potential confounders. Adjusted model **(B, D)**: analyses were adjusted for gestation age at birth, maternal age, body mass index (BMI), pregnancy-induced hypertension (PIH), gestational diabetes mellitus (GDM), and fetal sex.

We recorded an arched association between maternal TSH levels in the first trimester and neonatal birth weight in the LT4 supplementation group, either in the crude model (*P*=0.007) or in the adjusted model (*P*=0.004). Interestingly, maternal TSH levels had a significant positive association with neonatal birth weight when maternal TSH was roughly in the normal range, which transformed to a slight negative association for high TSH levels (**Figures 5C, D**). However, there was an inverse correlation between maternal TSH in the first trimester and birth weight in the control group (**Figures 5A, B**).

## Discussion

Pregnant women with a history of thyroidectomy were treated with supplementary LT4 throughout pregnancy. However, few studies have examined the association between maternal LT4 supplementation and neonatal growth after maternal thyroidectomy for thyroid cancer, and it is unclear how supplementary LT4 influences intrauterine fetal growth patterns. The results of this study showed that the level of FT4 in the LT4 supplementation group was significantly higher in both the first and third trimesters of pregnancy. Moreover, we observed a significant inverse association between maternal FT4 levels in the first trimester of pregnancy and neonatal birth weight, and a higher maternal FT4 level was associated with lower neonatal birth weight.

This is in line with the results of other studies in which maternal FT4 levels in early pregnancy are inversely associated with birth weight, and high FT4 levels are associated with an increased risk of SGA infants when maternal TSH and FT4 levels are both within the normal ranges (6, 13). Previous studies have also shown a comparable association between abnormal thyroid status and alterations in fetal growth patterns (10, 14, 15). Consistent with the current study, elevation in FT4 in euthyroid women contributes to lower birth weight. Haddow et al. (14) showed that euthyroid women with FT4 in the highest quintile gave birth to children with lower birth weight. Furthermore, pregnant women with hyperthyroidism were significantly associated with an increased risk of fetal growth restriction, preterm birth and low birth weight (15–17). Recently, a meta-analysis by Derahkshan et al. (18) also showed that a higher maternal FT4 concentration within the normal range is associated with lower birth weight and a higher risk of SGA, and this association suggests that levothyroxine therapy is associated with a potential risk of overtreatment, especially when targeting high-normal FT4 concentrations. Our findings suggest that sufficient LT4 during early pregnancy could maintain relatively high levels of intrauterine thyroxine by crossing the placenta. LT4 supplementation therapy, especially for TSH suppression therapy after thyroidectomy for thyroid cancer, may need to be monitored.

Thyroid hormone is a critical stimulator of fetal growth and development in early pregnancy and influences intrauterine fetal growth by stimulating fetal tissue metabolism. It is also necessary for fetal tissue accretion to trigger organ development (4). Fetal thyroxine availability during early pregnancy depends on the placental transfer of maternal thyroid hormones (4, 19). Maternal thyroid status is pivotal for the fetus in the first half of pregnancy, and maternal thyroid dysfunction affects fetal growth and birth weight (6). Previous studies found a consistent inverse association between maternal FT4 and estimated fetal growth, as well as head circumference and abdominal circumference (20), which suggests that maternal FT4 may play an important role in fetal soft tissue accretion and bone growth.

Birth weight is often used as a predictor of fetal growth and development. Fetal growth and weight reflect intrauterine conditions during pregnancy. Fetal growth during early pregnancy is a relevant determinant of pregnancy outcomes and later child health. However, it is unclear how supplementary LT4 after maternal thyroidectomy for thyroid cancer influences intrauterine fetal growth patterns. Interestingly, our results suggest that higher maternal FT4 levels during early pregnancy are associated with a significantly lower birth weight. The neonatal birth weight of women with LT4 supplementation was lower than that of control women, which could be partially explained by the fact that sufficient LT4 supplementation to suppress TSH may influence fetal growth. We also found a significant difference in the level of neonatal TSH between the two groups, and the neonatal TSH level was lower in the LT4 supplementation group than in the control group. This may be because the intrauterine thyroxine level could be high in the LT4 supplementation group, thereby causing lower TSH levels and lower birth weight.

The underlying physiological mechanism behind these observed associations between maternal FT4 levels and neonatal birth weight remains unclear. A potential mechanism may be related to maternal metabolic profile. A high normal FT4 level might act as a surrogate marker for reducing endogenous maternal glucose production, which would offer a possible explanation for the lower birth weight.

Casey et al. (21) found no difference in SGA between the LT4 and placebo groups in pregnant women with subclinical hypothyroidism or hypothyroxinemia when the reference criterion of LT4 supplementation was the TSH level in the normal range. In this study, we also did not find a significant difference in the risk of SGA between the LT4 supplementation group and the control group. Interestingly, a previous study showed a positive association between birth weight and cord TSH levels (6). We did not find a significant association between neonatal plantar blood TSH levels and birth weight, which may be attributed to the small sample size of patients with a history of thyroidectomy in this study.

A study showed that fetal sex also affects intrauterine fetal growth, and maternal thyroid metabolism may affect male fetuses to a greater extent (22). In our study, it appears that male fetuses suffer more from exposure to FT4 elevations, thereby leading to lower birth weight. However, it is borderline statistically significant, although we have adjusted the analysis by some potential confounders. One of the reasons could be attributed to some women not needing strict TSH suppression therapy, so the difference in FT4 levels between the two groups was reduced. Further studies are warranted to confirm these associations and to elucidate the underlying molecular mechanisms.

The strengths of our study include the large population-based study using maternal thyroid function parameters and neonatal birth weight. Moreover, we explored how thyroxine supplementation affects intrauterine fetal growth and the association between maternal LT4 supplementation after thyroidectomy for thyroid cancer and neonatal birth weight. Nevertheless, several potential limitations must be considered when interpreting these findings. First, although the included population of pregnant women was large, the number of patients who underwent thyroidectomy was relatively small. Second, we did not investigate the effects of maternal TPOAb positivity on birth weight. However, other studies have shown no association between maternal TPOAb positivity during early pregnancy and birth weight or fetal weight at mid and late pregnancy (6, 23). Third, detailed data on thyroid operation such as the extent of surgery, radioiodine therapy, and postoperative thyroid hormone replacement were lacking. However, the included patients underwent total thyroidectomy for thyroid cancer and required a sufficient dose of LT4 to replace or suppress TSH. In addition, although we adjusted for some potential confounders, other variables that could act as confounders were not included in the analyses, such as the detailed dose adjustment of LT4 supplementation, thyroid function test before pregnancy, and regional iodine status.

In summary, we investigated the association between maternal LT4 supplementation in early pregnancy and neonatal birth weight in women after thyroidectomy for thyroid cancer. These results are likely to raise concerns about maternal LT4 supplementation and variations in fetal growth patterns in women after surgery for thyroid cancer (24). Further studies are warranted to explore the biological mechanisms underlying this relationship.

## Conclusions

Taken together, we observed a significant influence of LT4 supplementation on neonatal birth weight in pregnant women with a history of thyroidectomy for thyroid cancer. The level of FT4 was higher and the neonatal birth weight was lower in the LT4 supplementation group than in the control group. We also observed a significant inverse association between maternal FT4 levels in early pregnancy and neonatal birth weight in the LT4 supplementation group. Based on these findings, maternal thyroid function and fetal ultrasound measurements should be monitored carefully during pregnancy in these women. Considering the limited number of available patients, further studies are warranted to strengthen these conclusions.

## Data Availability Statement

The original contributions presented in the study are included in the article/supplementary material. Further inquiries can be directed to the corresponding author.

## Ethics Statement

The study protocol was approved by the Medical Ethics Committee of the International Peace Maternity and Child Health Hospital. Written informed consent was obtained from all participants.

## Author Contributions

ZD analyzed the data and wrote the manuscript. FG and YZ took part in data management and analyses. XH collected the data. ZL reviewed the manuscript. JF designed the study and contributed to manuscript revision. All authors contributed to the article and approved the submitted version.

## Funding

This work was supported by the National Key Research and Development Program of China (2018YFC1004600).

## Conflict of Interest

The authors declare that the research was conducted in the absence of any commercial or financial relationships that could be construed as a potential conflict of interest.

## Publisher’s Note

All claims expressed in this article are solely those of the authors and do not necessarily represent those of their affiliated organizations, or those of the publisher, the editors and the reviewers. Any product that may be evaluated in this article, or claim that may be made by its manufacturer, is not guaranteed or endorsed by the publisher.

## References

[1] Smith LH Danielsen B Allen ME Cress R. Cancer Associated With Obstetric Delivery: Results of Linkage With the California Cancer Registry. Am J Obstet Gynecol (2003) 189:1128–35. doi: 10.1067/s0002-9378(03)00537-4 14586366

[2] Alexander EK Pearce EN Brent GA Brown RS Chen H Dosiou C. 2017 Guidelines of the American Thyroid Association for the Diagnosis and Management of Thyroid Disease During Pregnancy and the Postpartum. Thyroid (2017) 27(3):315–89. doi: 10.1089/thy.2016.0457 28056690

[3] Barjaktarovic M Korevaar TI Chaker L Jaddoe VWV Rijke YB Visser TJ. The Association of Maternal Thyroid Function With Placental Hemodynamics. Hum Reprod (2017) 32(3):653–61. doi: 10.1093/humrep/dew357 28130433

[4] Forhead AJ Fowden AL. Thyroid Hormones in Fetal Growth and Prepartum Maturation. J Endocrinol (2014) 221(3):R87–103. doi: 10.1530/JOE-14-0025 24648121

[5] Su PY Huang K Hao JH Xu YQ Yan SQ Li T. Maternal Thyroid Function in the First Twenty Weeks of Pregnancy and Subsequent Fetal and Infant Development: A Prospective Population-Based Cohort Study in China. J Clin Endocrinol Metab (2011) 96(10):3234–41. doi: 10.1210/jc.2011-0274 21832110

[6] Medici M Timmermans S Visser W Muinck Keizer-Schrama SMPF Jaddoe VWW Hofman A. Maternal Thyroid Hormone Parameters During Early Pregnancy and Birth Weight: The Generation R Study. J Clin Endocrinol Metab (2013) 98(1):59–66. doi: 10.1210/jc.2012-2420 23150694

[7] Korevaar TIM Medici M Visser TJ Peeters RP. Thyroid Disease in Pregnancy: New Insights in Diagnosis and Clinical Management. Nat Rev Endocrinol (2017) 13:610 –22. doi: 10.1038/nrendo.2017.93 28776582

[8] Bernal J. Thyroid Hormone Regulated Genes in Cerebral Cortex Development. J Endocrinol (2017) 232:R83–97. doi: 10.1530/JOE-16-0424 27852726

[9] Albu AR Anca AF Horhoianu VV Horhoianu IA. Predictive Factors for Intrauterine Growth Restriction. J Med Life (2014) 7(2):165–71. PMC4197512 25408721

[10] Shields BM Knight BA Hill A Hattersley AT Vaidya B. Fetal Thyroid Hormone Level at Birth Is Associated With Fetal Growth. J Clin Endocrinol Metab (2011) 96(6):E934–8. doi: 10.1210/jc.2010-2814 PMC3100744 21411545

[11] Cosmi E Fanelli T Visentin S Trevisanuto D Zanardo V. Consequences in Infants That Were Intrauterine Growth Restricted. J Pregnancy (2011) 2011:364381. doi: 10.1155/2011/364381 21547088 PMC3087146

[12] Risnes KR Vatten LJ Baker JL Jameson K Sovio U Kajantie E. Birthweight and Mortality in Adulthood: A Systematic Review and Meta-Analysis. Int J Epidemiol (2011) 40(3):647–61. doi: 10.1093/ije/dyq267 21324938

[13] Vrijkotte TG Hrudey EJ Twickler MB. Early Maternal Thyroid Function During Gestation Is Associated With Fetal Growth, Particularly in Male Newborns. J Clin Endocrinol Metab (2017) 102(3):1059–66. doi: 10.1210/jc.2016-3452 28359096

[14] Haddow JE Craig WY Neveux LM Haddow HRM Palomaki GE Lambert-Messerlian G. First and Second Trimester Risk of Aneuploidy (FaSTER) Research Consortium. Implications of High Free Thyroxine (FT4) Concentrations in Euthyroid Pregnancies: The FaSTER Trial. J Clin Endocrinol Metab (2014) 99(6):2038–44. doi: 10.1210/jc.2014-1053 PMC4037729 24606107

[15] León G Murcia M Rebagliato M Álvarez-Pedrerol M Castilla AM Basterrechea M. Maternal Thyroid Dysfunction During Gestation, Preterm Delivery, and Birthweight. The Infanciay Medio Ambiente Cohort, Spain. Paediatr Perinat Epidemiol (2015) 29(2):113–22. doi: 10.1111/ppe.12172 25565408

[16] Luewan S Chakkabut P Tongsong T. Outcomes of Pregnancy Complicated With Hyperthyroidism: A Cohort Study. Arch Gynecol Obstet (2011) 283:243–47. doi: 10.1007/s00404-010-1362-z 20087627

[17] Phoojaroenchanachai M Sriussadaporn S Peerapatdit T Vannasaeng S Nitiyanant W Boonnamsiri V. Effect of Maternal Hyperthyroidism During Late Pregnancy on the Risk of Neonatal Low Birth Weight. Clin Endocrinol (2001) 54:365–70. doi: 10.1046/j.1365-2265.2001.01224.x 11298089

[18] Derakhshan A Peeters RP Taylor PN Bliddal S Carty DM Meems M. Association of Maternal Thyroid Function With Birthweight: A Systematic Review and Individual-Participant Data Meta-Analysis. Lancet Diabetes Endocrinol (2020) 8(6):501–10. doi: 10.1016/S2213-8587(20)30061-9 PMC8168324 32445737

[19] Chan SY Vasilopoulou E Kilby MD. The Role of the Placenta in Thyroid Hormone Delivery to the Fetus. Nat Clin Pract Endocrinol Metab (2009) 5(1):45–54. doi: 10.1038/ncpendmet1026 19079273

[20] Johns LE Ferguson KK Cantonwine DE Mukherjee B Meeker JD McElrath TF. Subclinical Changes in Maternal Thyroid Function Parameters in Pregnancy and Fetal Growth. J Clin Endocrinol Metab (2018) 103(4):1349–58. doi: 10.1210/jc.2017-01698 PMC6018657 29293986

[21] Casey BM Thom EA Peaceman AM Varner MW Sorokin Y Hirtz DG. Treatment of Subclinical Hypothyroidism or Hypothyroxinemia in Pregnancy. N Engl J Med (2017) 376(9):815–25. doi: 10.1056/NEJMoa1606205 PMC5605129 28249134

[22] Aiken CE Ozanne SE. Sex Differences in Developmental Programming Models. Reprod (2013) 145(1):R1–R13. doi: 10.1530/REP-11-0489 23081892

[23] Männistö T Vääräsmäki M Pouta A Hartikainen AL Ruokonen A Surcel HM. Perinatal Outcome of Children Born to Mothers With Thyroid Dysfunction or Antibodies: A Prospective Population-Based Cohort Study. J Clin Endocrinol Metab (2009) 94:772–9. doi: 10.1210/jc.2008-1520 19106271

[24] Cho GJ Kim SY Lee HC Lee KM Han SW Oh MJ. Risk of Adverse Obstetric Outcomes and the Abnormal Growth of Offspring in Women With a History of Thyroid Cancer. Thyroid (2019) 29(6):879–85. doi: 10.1089/thy.2018.0283 PMC8917897 30957663

